# Risk of cardiovascular events associated with dipeptidyl peptidase-4 inhibitors in patients with diabetes with and without chronic kidney disease: A nationwide cohort study

**DOI:** 10.1371/journal.pone.0215248

**Published:** 2019-05-21

**Authors:** Tzu-Lan Huang, Fei-Yuan Hsiao, Chih-Kang Chiang, Li-Jiuan Shen, Chih-Fen Huang

**Affiliations:** 1 Graduate Institute of Clinical Pharmacy, College of Medicine, National Taiwan University, Taipei, Taiwan; 2 Department of Pharmacy, National Taiwan University Hospital, Taipei, Taiwan; 3 School of Pharmacy, College of Medicine, National Taiwan University, Taipei, Taiwan; 4 Graduate Institute of Toxicology, College of Medicine, National Taiwan University, Taipei, Taiwan; 5 Department of Integrated Diagnostics & Therapeutics, National Taiwan University Hospital, Taipei, Taiwan; Postgraduate Medical Institute, INDIA

## Abstract

**Background:**

Cardiovascular events associated with oral hypoglycemic agents (OHAs) have raised significant safety concerns. This study assessed the association between dipeptidyl peptidase-4 inhibitors (DPP-4i) and the risk of cardiovascular events in patients with type 2 diabetes mellitus with or without chronic kidney disease (CKD).

**Study design:**

A retrospective cohort study using Taiwan’s National Health Insurance Research Database.

**Settings and participants:**

Our study included patients with type 2 diabetes who received OHAs between March 1, 2009, and December 31, 2012. All eligible subjects were classified into CKD and non-CKD cohorts and further categorized as the DPP-4i and non-DPP-4i users in each cohort.

**Methods:**

The DPP-4i and non-DPP-4i groups were matched 1:1 by propensity score to attenuate potential selection bias. Propensity score was estimated by logistic regression, using demographics, co-medications, comorbidities. and adapted diabetic complication severity index at baseline.

**Outcomes:**

Outcomes of interest included a composite endpoint of ischemic stroke, myocardial infarction, cardiovascular death (major adverse cardiac events [MACE]), and hospitalization for heart failure (hHF). COX proportional hazard models were applied to examine the association between DPP-4i and outcomes of interest.

**Results:**

We identified 37,641 and 87,604 patients with type 2 diabetes with and without CKD, respectively. After propensity score matching, 8,213 pairs of CKD patients and 12,313 pairs of non-CKD patients were included for analysis. In the CKD cohort, DPP-4i were associated with a 25% increased risk of hHF (DPP-4i vs. non-DPP-4i incidence/1,000 person-years: 15.0 vs. 9.9, HR = 1.25; 95% CI 1.01–1.54, p = 0.037) but not with the risk of MACE (HR = 0.89, p = 0.144). In the non-CKD cohort, DPP-4i were associated with a lower risk of MACE (DPP-4i vs. non-DPP-4i incidence/1,000 person-years: 9.8 vs. 12.6 HR = 0.73; 95% CI 0.61–0.87, p = 0.0007), but not the risk of hHF (HR = 1.09, p = 0.631).

**Conclusions:**

DPP-4i were found to be associated with decreased risk of MACE in the non-CKD cohort in our study. However, DPP-4i were associated with increased risk of hHF in the CKD cohort. DPP-4i in the CKD cohort should be used cautiously.

## Introduction

Oral antidiabetic-agents-associated cardiovascular events have raised serious concerns since the debates about such risks among thiazolidinedione users that have arisen in the past decades[1]. As a result, the U.S. Food and Drug Administration /European Medical Association have requested cardiovascular safety trials with emerging antidiabetic agents, including Dipeptidyl peptidase-4 inhibitors (DPP-4i).

However, three major trials of DPP-4i have reported conflicting results. The SAVOR-TIMI-53, the first large randomized controlled safety trial aimed to assess the risk between DPP-4i and cardiovascular events, unexpectedly found a 27% increase in hospitalization for heart failure (hHF) in patients receiving saxagliptin compared to placebo[2]. However, results from the TECOS trial (sitagliptin) and EXAMINE trial (alogliptin) remained neutral regarding the risk of hHF in patients receiving DPP-4i [3, 4]. In addition, observational studies tried to answer this question and reported inconsistent findings[5–11].

Furthermore, despite the fact that numerous studies have explored the relationship between DPP-4i and cardiovascular events, studies that examined such risk among patients with diabetes and chronic kidney disease (CKD) remain scarce. Existing studies were often conducted in general diabetes populations, which include only a few CKD patients, if any[4–14]. The CKD subgroup had significant clinical relevance as the risk of cardiovascular events have been reported to be greater when patients develop both diabetes and CKD [15]. Other studies also suggested that diabetes and end-stage renal disease could synergistically increase risks of cardiovascular events—up to 5 times greater risk in some cases[16].

With limited available data, this study aims to assess the risk between DPP-4i and cardiovascular events in patients with type 2 diabetes with or without CKD. Particularly, we tested the hypothesis as to whether dialysis status or different DPP-4i contribute differently to our results.

## Methods

### Data source

We obtained healthcare data on patients with diabetes from the National Health Insurance Database (NHIRD). The National Health Insurance was a single-payer health insurance program initiated in 1995 and covered over 99% of total population in Taiwan. The NHIRD provides records on diagnoses, procedures, and drug prescriptions from the outpatient, inpatient, and emergency departments. For the current study, we used a subset of the NHIRD, the Longitudinal Health Insurance Database, which contains all the original claim data of 1 million beneficiaries randomly sampled from the NHIRD[17]. The study protocol was approved by the Research Ethics Committee of National Taiwan University Hospital (NTUH-REC-201406124W).

### Study cohort

All eligible patients had to have at least one diagnosis of diabetes mellitus (*International Classification of Diseases*, *Ninth Revision*, *Clinical Modification* [ICD-9-CM] code: 250) and at least one prescription of oral hypoglycemic agents (OHAs) between March 1, 2009 and December 31, 2012. The cohort identification period was defined as such because the first DPP-i was reimbursed in Taiwan since March 2009. Patients were excluded if they had type 1 diabetes (ICD-9-CM: 250.x1) and were under 20 years old upon cohort entry. We then divided the patients into two cohorts, the CKD group and non-CKD group, according to their underlying renal diseases (S2 Table) and dialysis status.

### Exposure assessment

Within the study cohort, patients with at least one prescription of DPP-4i during the cohort identification period were classified as the DPP-4i users, while all other were classified as the non-DPP-4i users. DPP-4i included in our study were sitagliptin, saxagliptin, vildagliptin, and linagliptin. To attenuate potential selection bias, we matched the non-DPP-4i users to the DPP-4i users using propensity score. We estimated the propensity score by logistic regression, using covariates, including age, gender, dialysis status, adapted diabetic complication severity index (aDCSI)[18–20], history of antidiabetic agent use, comorbidities, and selected co-medication use, and the greedy 5 to 1 technique was adopted for matching[21].

### Outcome measurement

The main outcomes of interest were hHF (ICD-9-CM: 428) and the composite endpoint of major adverse cardiovascular events (MACE), including myocardial infarction (ICD-9-CM: 410), ischemic stroke (ICD-9-CM: 433–435), and cardiovascular death. The definition of cardiovascular death meets the criteria of Standardized Definitions for End Point Events in Cardiovascular Trials draft by the U.S. Food and Drug Administration[22].

### Covariates

Baseline demographics included age, gender, dialysis status, aDCSI[18–20], history of antidiabetic agent use, comorbidities, and selected co-medication use. Baseline comorbidities were defined by diagnostic codes, and dialysis status was confirmed using data in the Registry for Catastrophic illness.

### Statistical analysis

Comparisons of demographics, comorbidities, and co-medications between the two user groups were conducted by paired statistical methods, paired t-tests for numeric variables, and McNemar’s test or Cochran’s Q test for categorical variables. All participants were followed until any events, death, end-of-follow-up, or if the patients dropped out of their original group (i.e., if they stop taking study medications, which mimics the as-treated analysis approach in clinical trials). Reentry into cohort was not allowed. Cox proportional hazard models were used to estimate hazard ratio (HR) with 95% confidence intervals (CIs).

## Results

**Fig 1:**
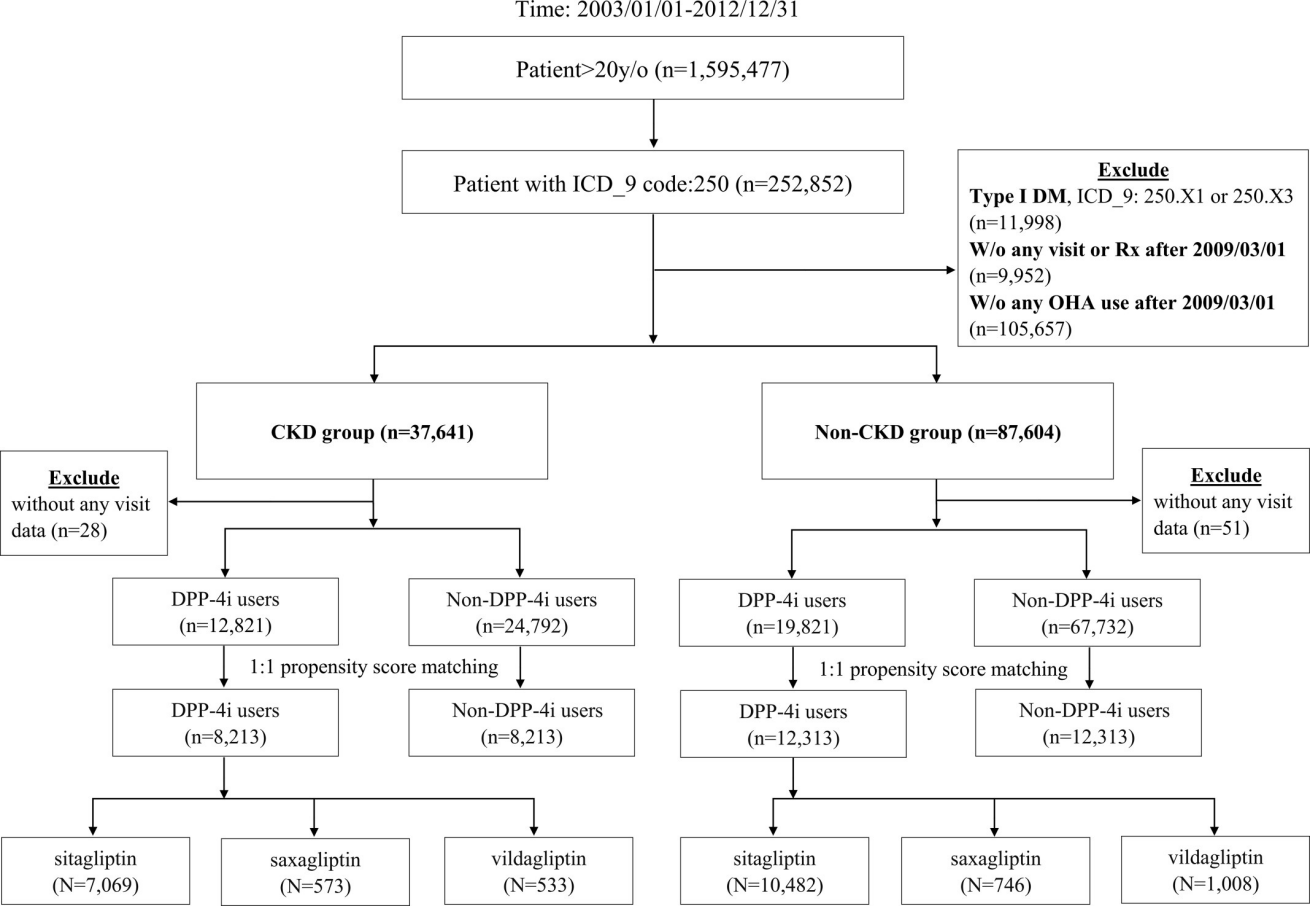
Cohort flow-chart and matching.

Between March 1, 2009, and December 31, 2012, we identified 8,213 pairs of CKD patients and 12,313 pairs of non-CKD patients after propensity-score matching. Baseline demographics were similar between the two user groups in both cohorts after matching (**Table 1, Fig 1**). Median follow-up for HF was 567 days (Q1-Q3: 176–1190) and 611 (Q1-Q3: 202–1298) days in the CKD and the non-CKD cohort, respectively. For MACE, the median follow-up was 553 days (Q1-Q3: 170–1161) and 600 (Q1-Q3: 196–1273) days in the CKD and the non-CKD cohort, respectively.

**Table 1:** Baseline characteristics of study cohort after matching.

Cohort/Group Characteristics	CKD population			Non-CKD population		
	DPP-4i	Non DPP-4i	P	DPP-4i	Non DPP-4i	P
**N**	8,213	8,213	-	12,313	12,313	-
Sex, male (%)	52.3	51.8	0.5184	50.2	50.4	0.8086
Age, year (mean)	65.7	65.7	0.8234	60.8	60.9	0.4163
Age, year (std)	12.6	11.9		12.7	11.9	
aDSCI (median, %)	2	2		1	1	
0	18.0	16.5	0.1176	48.8	48.7	0.6965
1	20.8	20.6		24.7	24.5	
2	22.0	21.7		16.1	16.5	
3	14.8	15.5		6.2	6.0	
4	11.8	12.5		3.1	2.9	
5+	12.7	13.2		1.3	1.4	
Dialysis status	9.9	10.6	0.1611	-	-	-
**Comorbidity, prior 24 months (%)**						
Lipid disorder	57.6	58.0	0.6569	55.3	55.0	0.7052
Obesity	1.3	1.3	0.9442	1.7	1.8	0.7302
Hypertension	78.1	78.0	0.8928	64.1	63.6	0.4002
Transplant	0.6	0.7	0.5507	0.1	0.1	0.8601
AMI*	3.4	3.7	0.3791	2.1	2.0	0.7849
CHF*	11.2	11.6	0.5023	4.3	4.4	0.7520
CHD	28.7	28.9	0.7680	20	19.7	0.5513
VHD*	3.2	3.2	1.0000	2.8	2.9	0.7308
AFib*	3.0	3.2	0.4972	1.6	1.7	0.6888
PAD*	12.6	12.8	0.6399	6.3	6.3	1.0000
Ischemic stroke	11.2	11.1	0.8614	7.3	7.6	0.4140
Anemia	0.6	0.7	0.7688	0.01	0.01	1.0000
PE*	0.1	0.2	0.8318	0.09	0.11	0.8388
Chronic lung disease	18.1	17.9	0.8063	12.9	13.3	0.3944
Cancer	8.3	8.4	0.9097	6.6	6.5	0.9177
Hypoglycemia	8.6	9.1	0.2232	3.1	3.0	0.5287
Liver cirrhosis	2.5	2.7	0.2961	1.8	1.9	0.5088
Thyroid disorder	4.4	4.6	0.4733	4.8	4.9	0.6772
Autoimmune disease	3.3	3.3	1.0000	2.5	2.5	0.9671
**History of Antidiabetics Agent Use, Prior 24 months (%)**						
α-glucosidase inhibitor	28.2	29.0	0.2254	20.6	19.7	0.0713
Biguanide	76.0	78.1	0.0011	82.7	81.5	<0.001
Meglitinide	19.2	19.1	0.9029	10.0	9.7	0.4769
TZD*	30.8	30.4	0.5439	25.1	24.4	0.1435
Sulfonylurea	76.7	78.7	0.0014	71.1	70.0	0.0348
Insulin	25.7	27.6	0.0036	11.24	11.1	0.7563
OHA* **Usage, At Index Date (%)**						
Median No.	2	2		2	2	
Monotherapy	27.4	26.8	< .0001	21.44	22.1	0.0064
Dual therapy	43.1	45.4		44.5	43.3	
Triple therapy	26.9	25.9		32.1	32.2	
>3 therapy	2.7	1.9		2.1	2.4	
**Co-medications (%)**						
CCB*	49.2	49.1	0.9126	37.6	37.3	0.6751
ACEI*	17.9	18.4	0.4448	12.7	12.9	0.6696
ARB*	49.4	50.0	0.4745	36.7	37.0	0.6561
β-blocker	35.5	35.5	0.9341	28.4	27.7	0.2047
α-blocker	7.4	8.0	0.2063	3.5	3.5	1.0000
Diuretics	65.5	65.4	0.8265	48.8	49.2	0.5860
Nitrate	15.4	16.2	0.1648	9.2	8.8	0.2564
Aspirin	34.6	35.1	0.4633	27.8	27.6	0.7162
Antiplatelet other than aspirin	20.8	20.6	0.7976	10.6	10.6	0.9832
Anticoagulant*	3.5	3.7	0.6131	1.6	1.7	0.8805
Statins	39.6	39.4	0.7541	36.2	35.7	0.4046
Fibrates	11.8	11.9	0.8452	10.0	10.0	0.6973
NSAIDs*	54.0	54.2	0.7894	53.4	53.3	0.8184
COX-2 inhibitors	6.6	6.7	0.7755	4.9	4.9	1.0000
EPO*	5.0	5.4	0.2237	-	-	-
Hyperkalemia/ hyperphosphatemia agents	2.6	2.9	0.3607	0.2	0.2	1.0000

* Abbreviations: AMI, acute myocardial infarction; CHF, congestive heart failure; VHD, valvular heart disease; Afib, atrial fibrillation; PAD, peripheral artery disease; PE, pulmonary embolism; TZD, thiazolidinedione; CCB, calcium channel blocker; ACEI, angiotensin-converting enzyme inhibitor; ARB, angiotensin-2 receptor blocker; NSAID, nonsteroidal anti-inflammatory drugs; Anticoagulant., anticoagulation agents; EPO, erythropoietin; aDCSI, adjusted Diabetes Complications Severity Index; OHA, oral hyperglycemic agent

### Risks of hHF and MACE

In the CKD cohort, exposure to DPP-4i were associated with a 25% increase risk of hHF (DPP-4i vs. non-DPP-4i incidence/1,000 person-years: 15.0 vs. 9.9, HR = 1.25; 95% CI 1.01–1.54, p = 0.037) but not with the risk of MACE (HR = 0.89, p = 0.144) (**Table 2**). When we further stratified the CKD cohort according to dialysis status (S2B Table), only those not undergoing dialysis showed an increased risk of hHF associated with DPP-4i use. However, the number of patients undergoing dialysis in this cohort (n = 1,687) was relatively small, which may lead to inadequate statistic power with only numeric increased risk of hHF (HR = 1.18, p = 0.48). Cardiovascular death showed a decreased risk toward DPP-4i, but the case number was very small (1 vs. 11) and therefore could not be further analyzed.

**Table 2:** Risks of hHF and MACE.

	CKD population						Non-CKD population					
	DPP-4i^b^		Non-DPP-4i				DPP-4i		Non-DPP-4i			
	N (%)	^a^	N (%)	^a^	HR (95% CI)	p-value	N (%)	^a^	N (%)	^a^	HR (95% CI)	p-value
**hHF**^b^	166 (2.0)	15.01	207 (2.5)	9.85	1.25 (1.01, 1.54)	0.0373	50 (0.4)	2.81	79 (0.6)	2.37	1.09 (0.76,1.59)	0.6314
**MACE**^b^	230 (2.8)	20.95	447 (5.4)	21.70	0.89 (0.75, 1.04)	0.1443	174 (1.4)	9.84	412 (3.3)	12.58	0.73 (0.61,0.87)	0.0007
MI^b^	72 (0.9)	6.48	129 (1.6)	6.11	0.98 (0.73, 1.32)	0.8982	44 (0.4)	2.47	85 (0.7)	2.55	1.00 (0.65, 1.40)	0.8105
Ischemic stroke	160 (1.9)	14.50	319 (3.9)	15.39	0.87 (0.72, 1.06)	0.1595	131 (1.1)	7.40	327 (2.7)	9.96	0.68 (0.55,0.84)	0.0003
CV death^b^	1 (<0.1)	0.09	11 (0.1)	0.52	0.11 (0.01, 0.82)	0.0318	2 (<0.01)	0.11	5 (<0.1)	0.15	0.42 (0.08, 2.17)	0.3017

^a^Incidence = number of events/ 1000 person-year

^b^Abbreviations: DPP-4i, DPP-4 inhibitors; hHF, hospitalization for heart failure; MACE, major adverse cardiovascular disease; MI, myocardial infarction; CV death, cardiovascular death

On the contrary, exposure to DPP-4i was associated with a lower risk of MACE (DPP-4i vs. non-DPP-4i incidence/1,000 person-years: 9.8 vs. 12.6, HR = 0.73; 95% CI 0.61–0.87, p = 0.0007), but not the risk of hHF (HR = 1.09, p = 0.631) in the non-CKD cohort (**Table 2**). The decreased risk of MACE in the non-CKD population is mainly contributed to the decrease risk of ischemic stroke (DPP-4i vs. non-DPP-4i incidence/1,000 person-years: 7.4 vs. 10.0, HR = 0.68; 95% CI 0.55–0.84, p = 0.0003) related to exposure of DPP-4i. No association between DPP-4i and decreased risk of the other two components of MACE were seen.

Overall, results were consistent in subgroup analysis stratified by age, gender, aDCSI, and other covariates (S3–S7 Tables).

### Subgroup analysis stratified by DPP-4i

When stratified by different DPP-4i use, the CKD group included 14,138, 1,146, and 1,066 participants in the sitagliptin, saxagliptin, and vildagliptin group, respectively. Similar distribution of patients was seen in the non-CKD group. Only sitagliptin showed consistent results with the main analysis (**Table 3**). Small case numbers of saxagliptin and vildagliptin led to wide CIs and made the results difficult to interpret.

**Table 3:** DPP-4 inhibitors subgroup analysis.

(a) *hHF subgroup analysis*				
**hHF**	**DPP-4i vs. Non-DPP-4i**			
	**All**	**Sitagliptin**	**Saxagliptin**	**Vildagliptin**
CKD population				
**N**	16,428	14,138	1,146	1,066
Events**	166/ 207	137/ 171	10/ 23	4/ 12
Person-year**	10004/ 20942	9087/ 17996	499/ 1534	418/ 1411
Incidence**	15.01/ 9.85	15.08/ 9.50	20.02/ 14.99	9.57/ 8.50
**HR (95% CI)**	1.25 (1.01, 1.54)	1.26 (1.00, 1.58)	1.08 (0.48, 2.42)	0.79 (0.24, 2.63)
**P-value**	0.0373	0.0480	0.8575	0.6979
Non-CKD population				
**N**	26,436	20,964	1,492	2,016
Events**	50/ 79	45/ 70	2/ 1	3/ 8
Person-year**	17353/ 33059	15706 / 28414	690/ 1999	956/ 2645
Incidence**	2.81/ 2.37	2.87/ 2.46	2.89/ 0.50	3.14/ 3.02
**HR (95% CI)**	1.09 (0.76, 1.58)	1.09 (0.73, 1.61)	4.31 (0.32, 57.72)	0.78 (0.18, 3.29)
**P-value**	0.6314	0.6706	0.2698	0.7314
(b) MACE subgroup analysis				
**MACE**	**DPP-4i vs. Non-DPP-4i**			
	**All**	**Sitagliptin**	**Saxagliptin**	**Vildagliptin**
CKD population
Events**	230/447	200/399	7/21	2/22
Person-year**	9932/ 20523	9014/ 17599	498/1528	419/ 1395
Incidence**	20.95/ 21.70	22.19/ 22.67	14.03/13.74	4.77/ 15.76
**HR (95% CI)**	0.89 (0.75, 1.04)	0.89 (0.74, 1.05)	0.92 (0.36, 2.35)	0.40 (0.09, 1.82)
**P-value**	0.1443	0.17	0.86	0.23
Non-CKD population
Events**	174/ 412	156/ 348	11/ 21	13/ 41
Person-year**	17213/ 32554	15574/ 27999	685/ 1960	953/ 2595
Incidence**	9.84/ 12.58	10.02/ 12.43	16.04/ 10.71	13.63/ 15.80
**HR (95% CI)**	0.73 (0.61, 0.87)	0.75 (0.62, 0.91)	1.12 (0.51, 2.46)	0.89 (0.44, 1.80)
**P-value**	0.0007	0.004	0.79	0.74

*Abbreviations: DPP-4i, DPP-4 inhibitors; hHF, hospitalization for heart failure; MACE, major adverse cardiovascular disease; CKD, chronic kidney disease

** Shown as DPP-4/ Non-DPP-4 group, incidence rates are expressed in events/ 1000-person-year

## Discussion

To the best of our knowledge, our study is the first to examine the association between DPP-4i and cardiovascular events in patients with type 2 diabetes with or without CKD. Compared to the non-DPP-4i users, DPP-4 significantly increased 25% risk of hHF in CKD patients. This is consistent with the SAVOR-TIMI-53 trial, which shows a 27% increased risk of hHF associated with saxagliptin use, regardless of kidney disease status[2]. Udell et al. stratified patients according to kidney function in their *post hoc* analysis and found that increased risk of hHF is seen only in patients with moderately impaired renal function (30 ml/min/1.73 m^2^ < estimated glomerular filtration rate [eGFR] <50 ml/min/1.73 m^2^), but not the normal-to-mildly impaired and the severely impaired renal function group (eGFR <30 ml/min/1.73 m^2^) [23]. Their results are similar to those of our study, since the increased risk is seen only in the CKD cohort but not the non-CKD cohort or dialysis subgroup. However, we have to bear in mind that the number of patients in the most severely impaired renal function group is small in both our study (i.e., dialysis subgroup) and the *post hoc* analysis. Another difference between our study and the *post hoc* analysis is that our patients were predominately sitagliptin users.

In another observational study done in Taiwan, Ou et al. found no association between DPP-4i and hHF, whether or not the patients had CKD[6]. However, Ou et al.’s comparator groups are different from ours, and they analyzed the CKD group by using subgroup analysis instead of matched cohort, which we emphasized to counter the action of selection bias. Our work was also consistent with another study, which specifically focused on the dialysis patients and found no increased risk between DPP-4i and hHF (HR = 1.14, 95% CI 0.85–1.54)[24]. DPP-4i are a class of drug with pleiotropic effects, and the mechanism between DPP-4i and heart failure is unclear. In an animal model, selectively using DPP-4i in old, diabetic mice results in modest cardiac hypertrophy, impairment of cardiac function, and dysregulated expression of genes and proteins controlling inflammation and cardiac fibrosis[25]. All these findings were not seen in the young DPP4 knockout mice. Further mechanistic experimental studies were warranted to explore the myth of DPP4i in the risk of hHF in different patient characteristics.

Although there was no association between DPP-4i and MACE in all three clinical trials, our study shows that DPP-4i were associated with decreased risk of MACE in the non-CKD cohort. When broken down to specific components, DPP-4i use is associated with a 32% decrease in risk of ischemic stroke. These results are similar to those of most of the observational studies done in Taiwan[5, 6, 24]. However, in Ou et al.’s study, DPP-4i decreased risk of ischemic stroke regardless of kidney disease status[6]. More and more studies have been showing that DPP-4 inhibition exhibit neuroprotective effect, either through glucagon-like peptide-mediated or other pathways[26, 27].

Our studies have the following strengths in answering a significant clinical question. First, using the Longitudinal Health Insurance Database, we have a very large sample size (N = 42,864) of unselected patient coverage, which would fit to the general practices of care of patients with type 2 diabetes. Unlike many others, our study population is not restricted to patients with newly diagnosed disease or patients using certain combination of OHA only, which more closely resembles real-world practice. Second, although we are unable to differentiate the effects of saxagliptin, sitagliptin, vildagliptin, and linagliptin on cardiovascular events, we include all four DPP-4i available. Thirdly, our study’s follow-up period is relatively long, with medians of 1.3 and 1.6 years in different models. Finally, we demonstrated the differences between CKD and non-CKD patients, stratified patients into two matched groups, and analyzed them in similar fashion.

However, our study has several limitations, which are similar to those of other observational studies based on claims databases. Firstly, due to the lack of laboratory data and other information in NHIRD, we cannot adjust for confounders such as creatinine clearance, HbA1c, or information such as smoking status, body mass index, and other socioeconomic factors, which may lead to unbalanced characteristics between groups and generate bias. However, we applied aDCSI, history of antidiabetic agent use as diabetes severity markers, erythropoietin (EPO) use, and anemia percentage as CKD severity markers. We found only minor differences in CKD and diabetes duration between the treatment groups. Secondly, although the diabetes definition[28–30], CKD definition,[31–33] and outcome codes we used in the study have been validated in NHIRD or large databases, misclassification of events or drug exposure may still exist when conducting database research. As-treated analysis may be able to minimize exposure misclassifications. Thirdly, drug exposure definition relied solely on prescriptions; therefore, patient adherence is unknown and uncontrolled. Another limitation in the study is the possibility of CKD case misclassification; since our screening period of CKD followed those with diabetes, some CKD group patients may not have developed CKD upon cohort entry. However, after excluding those patients and their matched participants, the results remain consistent with those of the main analysis (hHF, HR = 1.54, p = 0.001; MACE, HR = 0.93, p = 0.49). Fourthly, amongst the various DPP-4is, linagliptin is currently being used quite frequently in CKD patients; however, linagliptin was not reimbursed by Taiwan’s National Health Insurance program until June, 2012. As our study cohort was identified as those with diagnosis of diabetes mellitus and received prescription of OHAs between March 1, 2009 and December 31, 2012, less than 20 linagliptin users were included in our study. The low number of participants make it implausible to analyze linaglipin solely. Further studies evaluating the CV profiles of linagliptin are thus warranted.

## Conclusion

For patients with type 2 diabetes without CKD, we found that DPP-4i exposure is associated with a lower risk of MACE and ischemic stroke. However, DPP-4i are associated with a higher risk of hHF in patients with type 2 diabetes with CKD. DPP-4i in the CKD cohort should be used cautiously. Future studies with more comprehensive hospital-based cohorts with more detailed patient data are warranted.

## Supporting information

S1 Table OHA drug lists and ATC codes. (DOCX)

S2 Table CKD definition: Diagnosis codes and dialysis codes. (a) CKD-related diagnosis codes (b) Dialysis codes.
(DOCX)

S3 Table Subgroup analysis-hHF in CKD population. (DOCX)

S4 Table Subgroup analysis-hHF in non-CKD population. (DOCX)

S5 Table Subgroup analysis-MACE in CKD population. (DOCX)

S6 Table Subgroup analysis-MACE in non-CKD population. (DOCX)

S7 Table Subgroup analysis-ischemic stroke in non-CKD population. (DOCX)

## References

[1] Nissen SE, Wolski K. Effect of rosiglitazone on the risk of myocardial infarction and death from cardiovascular causes. N Engl J Med. 2007;356(24):2457–71. 10.1056/NEJMoa072761 . 17517853

[2] Scirica BM, Bhatt DL, Braunwald E, Steg PG, Davidson J, Hirshberg B, et al Saxagliptin and cardiovascular outcomes in patients with type 2 diabetes mellitus. N Engl J Med. 2013;369(14):1317–26. 10.1056/NEJMoa1307684 . 23992601

[3] Zannad F, Cannon CP, Cushman WC, Bakris GL, Menon V, Perez AT, et al Heart failure and mortality outcomes in patients with type 2 diabetes taking alogliptin versus placebo in EXAMINE: a multicentre, randomised, double-blind trial. Lancet. 2015;385(9982):2067–76. 10.1016/S0140-6736(14)62225-X . 25765696

[4] Green JB, Bethel MA, Armstrong PW, Buse JB, Engel SS, Garg J, et al Effect of Sitagliptin on Cardiovascular Outcomes in Type 2 Diabetes. N Engl J Med. 2015;373(3):232–42. 10.1056/NEJMoa1501352 . 26052984

[5] Yang TY, Liaw YP, Huang JY, Chang HR, Chang KW, Ueng KC. Association of Sitagliptin with cardiovascular outcome in diabetic patients: a nationwide cohort study. Acta Diabetol. 2016;53(3):461–8. 10.1007/s00592-015-0817-x . 26687195

[6] Ou SM, Shih CJ, Chao PW, Chu H, Kuo SC, Lee YJ, et al Effects on Clinical Outcomes of Adding Dipeptidyl Peptidase-4 Inhibitors Versus Sulfonylureas to Metformin Therapy in Patients With Type 2 Diabetes Mellitus. Ann Intern Med. 2015;163(9):663–72. 10.7326/M15-0308 . 26457538

[7] Chang YC, Chuang LM, Lin JW, Chen ST, Lai MS, Chang CH. Cardiovascular risks associated with second-line oral antidiabetic agents added to metformin in patients with Type 2 diabetes: a nationwide cohort study. Diabet Med. 2015;32(11):1460–9. 10.1111/dme.12800 . 25970814

[8] Chen DY, Wang SH, Mao CT, Tsai ML, Lin YS, Chou CC, et al Sitagliptin and cardiovascular outcomes in diabetic patients with chronic kidney disease and acute myocardial infarction: A nationwide cohort study. Int J Cardiol. 2015;181:200–6. 10.1016/j.ijcard.2014.12.029 . 25528312

[9] Wang KL, Liu CJ, Chao TF, Huang CM, Wu CH, Chen SJ, et al Sitagliptin and the risk of hospitalization for heart failure: a population-based study. Int J Cardiol. 2014;177(1):86–90. 10.1016/j.ijcard.2014.09.038 . 25499347

[10] Chen DY, Wang SH, Mao CT, Tsai ML, Lin YS, Su FC, et al Sitagliptin After Ischemic Stroke in Type 2 Diabetic Patients: A Nationwide Cohort Study. Medicine (Baltimore). 2015;94(28):e1128 10.1097/MD.0000000000001128 26181549 PMC4617065

[11] Wang SH, Chen DY, Lin YS, Mao CT, Tsai ML, Hsieh MJ, et al Cardiovascular Outcomes of Sitagliptin in Type 2 Diabetic Patients with Acute Myocardial Infarction, a Population-Based Cohort Study in Taiwan. PLoS One. 2015;10(6):e0131122 10.1371/journal.pone.0131122 26115092 PMC4482692

[12] Ingelheim B. CAROLINA: Cardiovascular Outcome Study of Linagliptin Versus Glimepiride in Patients With Type 2 Diabetes. Available from: https://clinicaltrials.gov/ct2/show/NCT01243424 NLM Identifier: NCT01243424.

[13] Ingelheim B. Cardiovascular and Renal Microvascular Outcome Study With Linagliptin in Patients With Type 2 Diabetes Mellitus (CARMELINA).

[14] White WB, Cannon CP, Heller SR, Nissen SE, Bergenstal RM, Bakris GL, et al Alogliptin after acute coronary syndrome in patients with type 2 diabetes. N Engl J Med. 2013;369(14):1327–35. 10.1056/NEJMoa1305889 . 23992602

[15] Go AS, Mozaffarian D, Roger VL, Benjamin EJ, Berry JD, Borden WB, et al Heart disease and stroke statistics—2013 update: a report from the American Heart Association. Circulation. 2013;127(1):e6–e245. 10.1161/CIR.0b013e31828124ad . 23239837 PMC5408511

[16] Chang YT, Wu JL, Hsu CC, Wang JD, Sung JM. Diabetes and end-stage renal disease synergistically contribute to increased incidence of cardiovascular events: a nationwide follow-up study during 1998–2009. Diabetes Care. 2014;37(1):277–85. 10.2337/dc13-0781 . 23920086

[17] Hsiao F-Y. Using Taiwan's National Health Insurance Research Databases for Pharmacoepidemiology Research. Journal of Food and Drug Analysis. 2007;15:99–108.

[18] Chang HY, Weiner JP, Richards TM, Bleich SN, Segal JB. Validating the adapted Diabetes Complications Severity Index in claims data. Am J Manag Care. 2012;18(11):721–6. . 23198714

[19] Chen HL, Hsiao FY. Risk of hospitalization and healthcare cost associated with Diabetes Complication Severity Index in Taiwan's National Health Insurance Research Database. J Diabetes Complications. 2014;28(5):612–6. 10.1016/j.jdiacomp.2014.05.011 . 25037987

[20] Young BA, Lin E, Von Korff M, Simon G, Ciechanowski P, Ludman EJ, et al Diabetes complications severity index and risk of mortality, hospitalization, and healthcare utilization. Am J Manag Care. 2008;14(1):15–23. 18197741 PMC3810070

[21] Parsons L. SUGI 26: Reducing Bias in a Propensity Score Matched-Pair Sample Using Greedy Matching Techniques. The SAS Institute 2001 2007.

[22] Hicks KA, Tcheng JE, Bozkurt B, Chaitman BR, Cutlip DE, Farb A, et al 2014 ACC/AHA Key Data Elements and Definitions for Cardiovascular Endpoint Events in Clinical Trials: A Report of the American College of Cardiology/American Heart Association Task Force on Clinical Data Standards (Writing Committee to Develop Cardiovascular Endpoints Data Standards). Circulation. 2015;132(4):302–61. 10.1161/CIR.0000000000000156 . 25547519

[23] Udell JA, Bhatt DL, Braunwald E, Cavender MA, Mosenzon O, Steg PG, et al Saxagliptin and cardiovascular outcomes in patients with type 2 diabetes and moderate or severe renal impairment: observations from the SAVOR-TIMI 53 Trial. Diabetes Care. 2015;38(4):696–705. 10.2337/dc14-1850 . 25552421

[24] Chan SY, Ou SM, Chen YT, Shih CJ. Effects of DPP-4 inhibitors on cardiovascular outcomes in patients with type 2 diabetes and end-stage renal disease. Int J Cardiol. 2016;218:170–5. 10.1016/j.ijcard.2016.05.062 . 27236110

[25] Mulvihill EE, Varin EM, Ussher JR, Campbell JE, Bang KW, Abdullah T, et al Inhibition of Dipeptidyl Peptidase-4 Impairs Ventricular Function and Promotes Cardiac Fibrosis in High Fat-Fed Diabetic Mice. Diabetes. 2016;65(3):742–54. 10.2337/db15-1224 . 26672095

[26] Darsalia V, Ortsater H, Olverling A, Darlof E, Wolbert P, Nystrom T, et al The DPP-4 inhibitor linagliptin counteracts stroke in the normal and diabetic mouse brain: a comparison with glimepiride. Diabetes. 2013;62(4):1289–96. 10.2337/db12-0988 23209191 PMC3609599

[27] Shannon RP. DPP-4 inhibition and neuroprotection: do mechanisms matter? Diabetes. 2013;62(4):1029–31. 10.2337/db12-1794 23520281 PMC3609569

[28] Lin CC, Lai MS, Syu CY, Chang SC, Tseng FY. Accuracy of diabetes diagnosis in health insurance claims data in Taiwan. J Formos Med Assoc. 2005;104(3):157–63. . 15818428

[29] Wilson C, Susan L, Lynch A, Saria R, Peterson D. Patients with diagnosed diabetes mellitus can be accurately identified in an Indian Health Service patient registration database. Public Health Rep. 2001;116(1):45–50. 10.1016/S0033-3549(04)50021-3 11571407 PMC1497292

[30] Zgibor JC, Orchard TJ, Saul M, Piatt G, Ruppert K, Stewart A, et al Developing and validating a diabetes database in a large health system. Diabetes Res Clin Pract. 2007;75(3):313–9. 10.1016/j.diabres.2006.07.007 . 16934906

[31] Kern EF, Maney M, Miller DR, Tseng CL, Tiwari A, Rajan M, et al Failure of ICD-9-CM codes to identify patients with comorbid chronic kidney disease in diabetes. Health Serv Res. 2006;41(2):564–80. 10.1111/j.1475-6773.2005.00482.x 16584465 PMC1702507

[32] Vlasschaert ME, Bejaimal SA, Hackam DG, Quinn R, Cuerden MS, Oliver MJ, et al Validity of administrative database coding for kidney disease: a systematic review. Am J Kidney Dis. 2011;57(1):29–43. 10.1053/j.ajkd.2010.08.031 . 21184918

[33] Winkelmayer WC, Schneeweiss S, Mogun H, Patrick AR, Avorn J, Solomon DH. Identification of individuals with CKD from Medicare claims data: a validation study. Am J Kidney Dis. 2005;46(2):225–32. 10.1053/j.ajkd.2005.04.029 . 16112040

