# Supplementary material for: Risk of cardiovascular events associated with dipeptidyl peptidase-4 inhibitors in patients with diabetes with and without chronic kidney disease: A nationwide cohort study
Source: PLoS One. 2019 May 21;14(5):e0215248. doi: 10.1371/journal.pone.0215248 (PMC6528980; doi:10.1371/journal.pone.0215248)
Supplement: S1 Table — (DOCX) [file pone.0215248.s001.docx]

**S1. OHA drug lists and ATC codes**

| Category | Name | ATC code |
| --- | --- | --- |
| alpha-glucosidase inhibitors | acarbose | A10BF01 |
|  | miglitol | A10BF02 |
| biguanide | buformin | A10BA03 |
|  | metformin hcl | A10BA02 |
| dipeptidyl peptidase-4  (DPP-4) inhibitors | linagliptin | A10BH05 |
|  | saxagliptin | A10BH03 |
|  | sitagliptin | A10BH01 |
|  | vildagliptin | A10BH02 |
| meglitinide | nateglinide | A10BX03 |
|  | repaglinide | A10BX02 |
| sulfonylurea (SFU) | acetohexamide | A10BB31 |
|  | chlorpropamide | A10BB02 |
|  | glibornuride | A10BB04 |
|  | gliclazide | A10BB09 |
|  | glimepiride | A10BB12 |
|  | glipizide | A10BB07 |
|  | gliquidone | A10BB08 |
|  | glyburide | A10BB01 |
|  | tolazamide | A10BB05 |
|  | tolbutamide | A10BB03 |
| thiazolidinedione  (TZD) | pioglitazone | A10BG03 |
|  | rosiglitazone | A10BG02 |
