# Supplementary material for: Risk of cardiovascular events associated with dipeptidyl peptidase-4 inhibitors in patients with diabetes with and without chronic kidney disease: A nationwide cohort study
Source: PLoS One. 2019 May 21;14(5):e0215248. doi: 10.1371/journal.pone.0215248 (PMC6528980; doi:10.1371/journal.pone.0215248)
Supplement: S2 Table — (a) CKD-related diagnosis codes (b) Dialysis codes. (DOCX) [file pone.0215248.s002.docx]

**S2. CKD definition: diagnosis codes and dialysis codes**

(a) CKD-related diagnosis codes

| ICD-9-CM | Disease name |
| --- | --- |
| 250.4 | diabetes with renal manifestations |
| 274.1 | gouty nephropathy |
| 283.11 | nonautoimmune hemolytic anemias, hemolytic-uremic syndrome |
| 403.x1 | hypertensive heart disease + renal failure |
| 404.x2 | hypertensive heart disease + renal failure |
| 404.x3 | hypertensive heart disease + renal failure + heart failure |
| 440.1 | atherosclerosis of renal artery |
| 442.1 | aneurysm of renal artery |
| 447.3 | hyperplasia of renal artery |
| 572.4 | hepatorenal syndrome |
| 585 | chronic renal failure |
| 586 | renal failure, unspecified |
| 587 | renal sclerosis, unspecified |
| 588 | disorders resulting from impaired renal function |
| 642.1 | hypertension secondary to renal disease, complicating pregnancy, childbirth, and puerperium |
| 642.2 | unspecified renal disease in pregnancy, without mention of hypertension |
| V451 | renal dialysis status |

*Abbreviation: CKD, chronic kidney disease

(b) Dialysis codes

| Databases | Columns | Codes |
| --- | --- | --- |
| Registry for catastrophic illness patients (HV) | HV_TYPE | 04 |
| Details of ambulatory care orders (OO) | CASE_TYPE | 05 |
