# Supplementary material for: Risk of cardiovascular events associated with dipeptidyl peptidase-4 inhibitors in patients with diabetes with and without chronic kidney disease: A nationwide cohort study
Source: PLoS One. 2019 May 21;14(5):e0215248. doi: 10.1371/journal.pone.0215248 (PMC6528980; doi:10.1371/journal.pone.0215248)
Supplement: S5 Table — (DOCX) [file pone.0215248.s005.docx]

**S5. Subgroup analysis-MACE in CKD population**


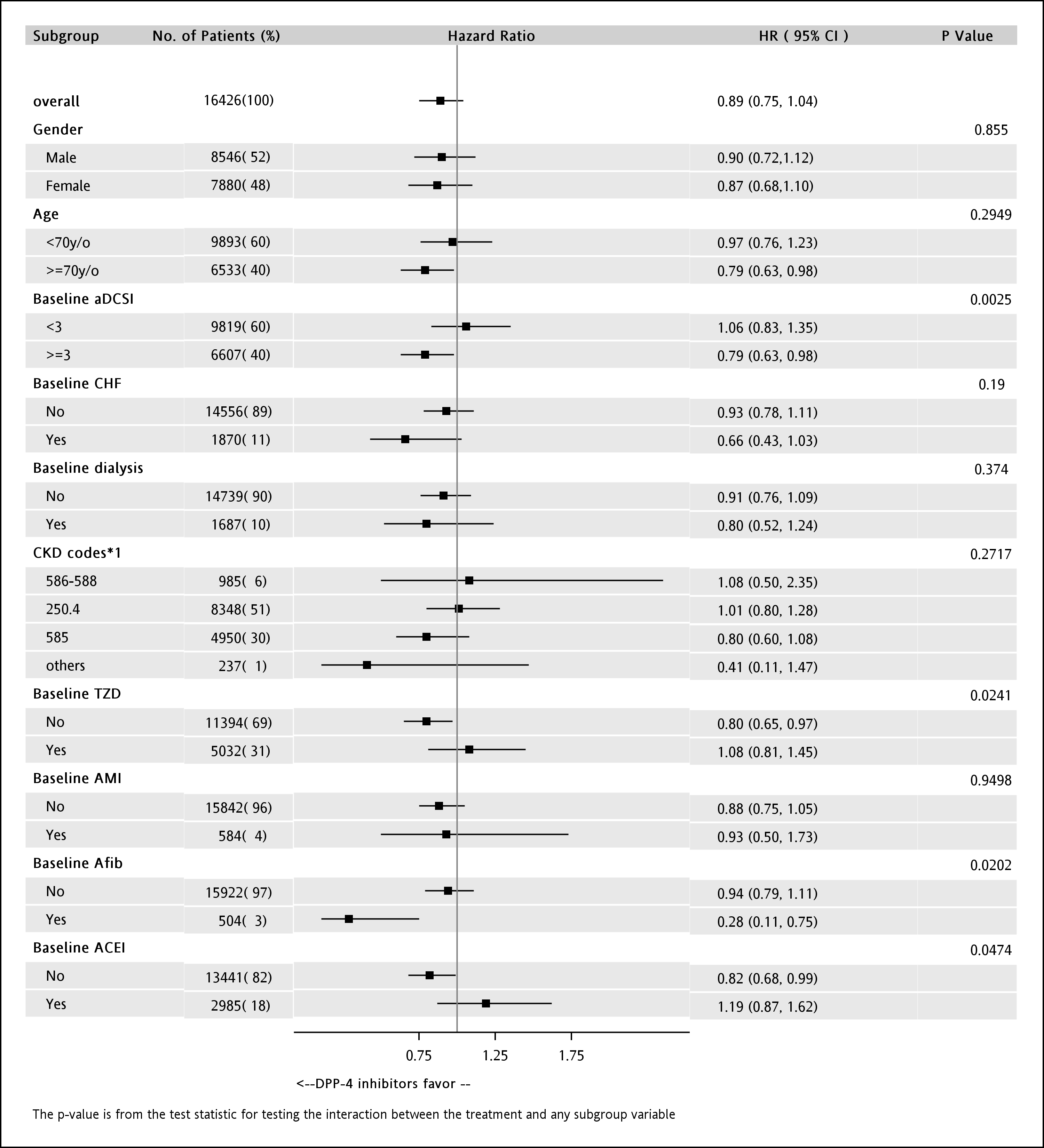


*Abbreviations: CKD, chronic kidney disease; DPP-4 inhibitors; hHF, hospitalization for heart failure; MACE, major adverse cardiovascular disease; CHF, congestive heart failure; aDCSI; TZD, thiazolidinedione; AMI, acute myocardial infarction; AFib, atrial fibrillation; ACEI, angiotensin-converting enzyme inhibitor; aDCSI, adjusted Diabetes Complications Severity Index

*1: 250.4: diabetes with renal manifestations; 274.1: gouty nephropathy; 585: chronic renal failure; 586: renal failure, unspecified; 587: renal sclerosis, unspecified; 588: disorders resulting from impaired renal function; others: please refer to table S2.
