# Supplementary material for: Risk of cardiovascular events associated with dipeptidyl peptidase-4 inhibitors in patients with diabetes with and without chronic kidney disease: A nationwide cohort study
Source: PLoS One. 2019 May 21;14(5):e0215248. doi: 10.1371/journal.pone.0215248 (PMC6528980; doi:10.1371/journal.pone.0215248)
Supplement: S7 Table — (DOCX) [file pone.0215248.s007.docx]

**S7. Subgroup analysis-ischemic stroke in non-CKD population**


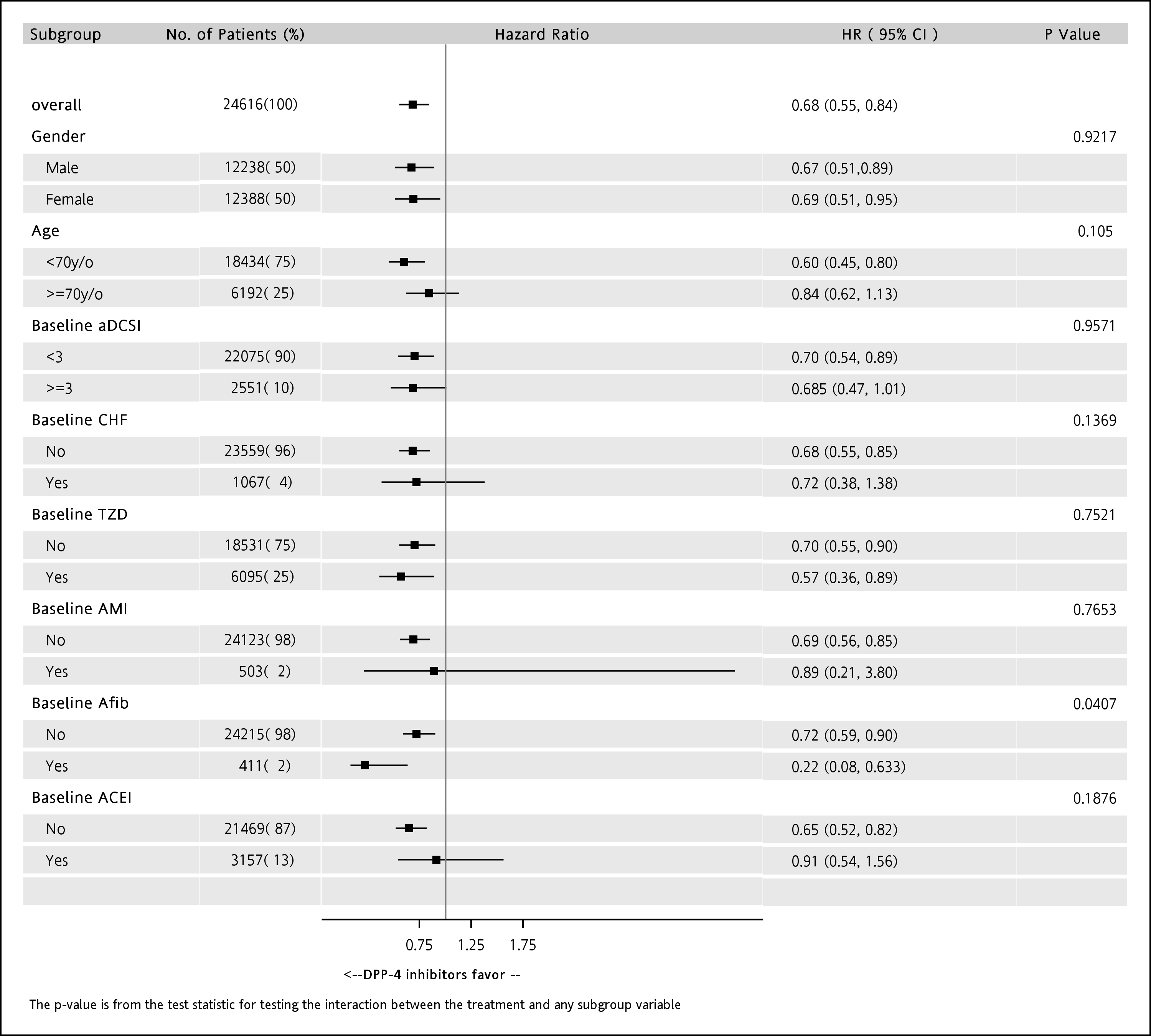


*Abbreviations: CKD, chronic kidney disease; DPP-4 inhibitors; hHF, hospitalization for heart failure; MACE, major adverse cardiovascular disease; CHF, congestive heart failure; aDCSI; TZD, thiazolidinedione; AMI, acute myocardial infarction; AFib, atrial fibrillation; ACEI, angiotensin-converting enzyme inhibitor; aDCSI, adjusted Diabetes Complications Severity Index
